# Neurotoxicity of microplastics: a CiteSpace-based review and emerging trends study

**DOI:** 10.1007/s10661-023-11559-1

**Published:** 2023-07-15

**Authors:** Zeyan Ye, Tingyu Mai, Yuqian Cheng, Xiashuang Zhang, Zhe Liu, Zhiyong Zhang, You Li

**Affiliations:** 1 Department of Environmental Health and Occupational Medicine, School of Public Health, Guilin Medical University, Zhiyuan Road No.1, Guilin, 541199 Guangxi Province People’s Republic of China; 2 The Guangxi Key Laboratory of Environmental Exposomics and Entire Lifecycle Heath, Guilin, People’s Republic of China

**Keywords:** Microplastic, Neurotoxicity, Visualization, Bibliometric, CiteSpace

## Abstract

Microplastics, as a currently emerging pollutant, are gaining increasing attention from researchers in various fields. The purpose of this study is to summarize research development on microplastics in the field of neurotoxicity using bibliometric tools and visualization methods and to identify current research hotspots. The Web of Science Core Collection (WoSCC) was searched under the topics of “microplastics” and “neurotoxicity.” A total of 33 published articles were obtained by exclusion and analyzed using CiteSpace (V6.1.R2). It was found that microplastic neurotoxicity research is currently on the rise, with the most research results being published in China, the most collaboration occurring between Italy and other countries, and the least collaboration occurring between authors. The focus and hotspots of future research on the neurotoxicity of microplastics may revolve around “accumulation” and “integrated biomarker response.” These findings demonstrate the trends and frontiers in the field of microplastic neurotoxicity research and provide valuable information for subsequent research directions and potential collaborations.

## Introduction

Plastic pollution is currently one of the most significant environmental pollution problems. Since microplastics was first defined by Thompson in 2004 (Thompson et al., 2004), they have been widely studied worldwide as an emerging pollutant in the environment (Lambert & Wagner, 2016; Mitrano et al., 2021). Microplastics are defined as small pieces of plastic particles of less than 5 mm in size that are widely distributed in the human environment in different sizes and shapes, polymers, and concentrations (Ren et al., 2021). Geyer et al. predicted that in the next 30 years, approximately 12 million tons of plastic will be released into landfills and our living environments (Geyer et al., 2017). Microplastics can enter the human body through the degradation of common plastic pollutants, agricultural mulch, sewage irrigation, air inhalation, atmospheric deposition, etc., causing damage to the body (Ren et al., 2021). The toxicity of microplastics has been studied in many systems and organs, including the digestive, immune, and reproductive systems, and toxic effects on the nervous system cannot be ignored (Deng et al., 2017; Jin et al., 2021; Stock et al., 2019; Yu et al., 2022).

CiteSpace not only allows one to explore and visualize the overall research content of a specialized field and its dynamics over time but also to discover important topics, authors, and published literature in the field (Chen & Leydesdorff, 2014). Microplastics are an emerging pollutant of focus in the current research (Jin et al., 2022). Therefore, the purpose of this paper is to clearly summarize and visualize the research development regarding microplastics in the field of neurotoxicity and to identify current research hotspots and provide suggestions and directions for future research in this field.

## Methods

### Data source

All data were retrieved on December 1, 2022, from the Web of Science Core Collection (WoSCC). The literature search formula was set as follows: (Topic Search = “microplastic” OR Topic Search = “micro-plastic” OR Topic Search = “Microplastic”) AND Topic Search = “Neurotoxicity.” To avoid duplication of analysis, review papers were excluded, and the time of publication was not limited. The titles and abstracts of the retrieved documents were read individually to ensure that the topics included met the requirements, and 33 documents were finally adopted.

The “full record and cited references” of the above documents in “plain text file” format were exported via the WoSCC. CiteSpace software was used to remove duplicate articles based on the given “title.” Finally, 33 postfiltered papers were obtained and included in the follow-up analysis study.

### Data analysis

The number of study objectives in the literature and trends over time were described and presented using Excel (2021). CiteSpace (V6.1. R2) was used to convert and deduplicate the formats of the included documents; calculate the centrality of authors, keywords, countries, and cocited references; draw author, country, and keyword cooccurrence maps, keyword clustering, and time zone view maps; and draw burst maps of keywords and cocited references.

The CiteSpace Years per Slice function was set to “1 year,” and different objects were selected. The mapping pruning algorithm was selected as the “Pathfinder” algorithm along with “Pruning slice networks.” Keywords clustering was conducted using the log-likelihood ratio (LLR) method. The most representative keywords of this type of group are selected as the label of this type of group. The size of the nodes in the graph reflects their count relative to other nodes in the graph, and the nodes are marked by purple circles indicating that these nodes have a high (not less than 0.1) centrality. The color of a node linkage corresponds to the time year shown in the legend above the map.

## Results

### Growth trends

**Fig. 1:**
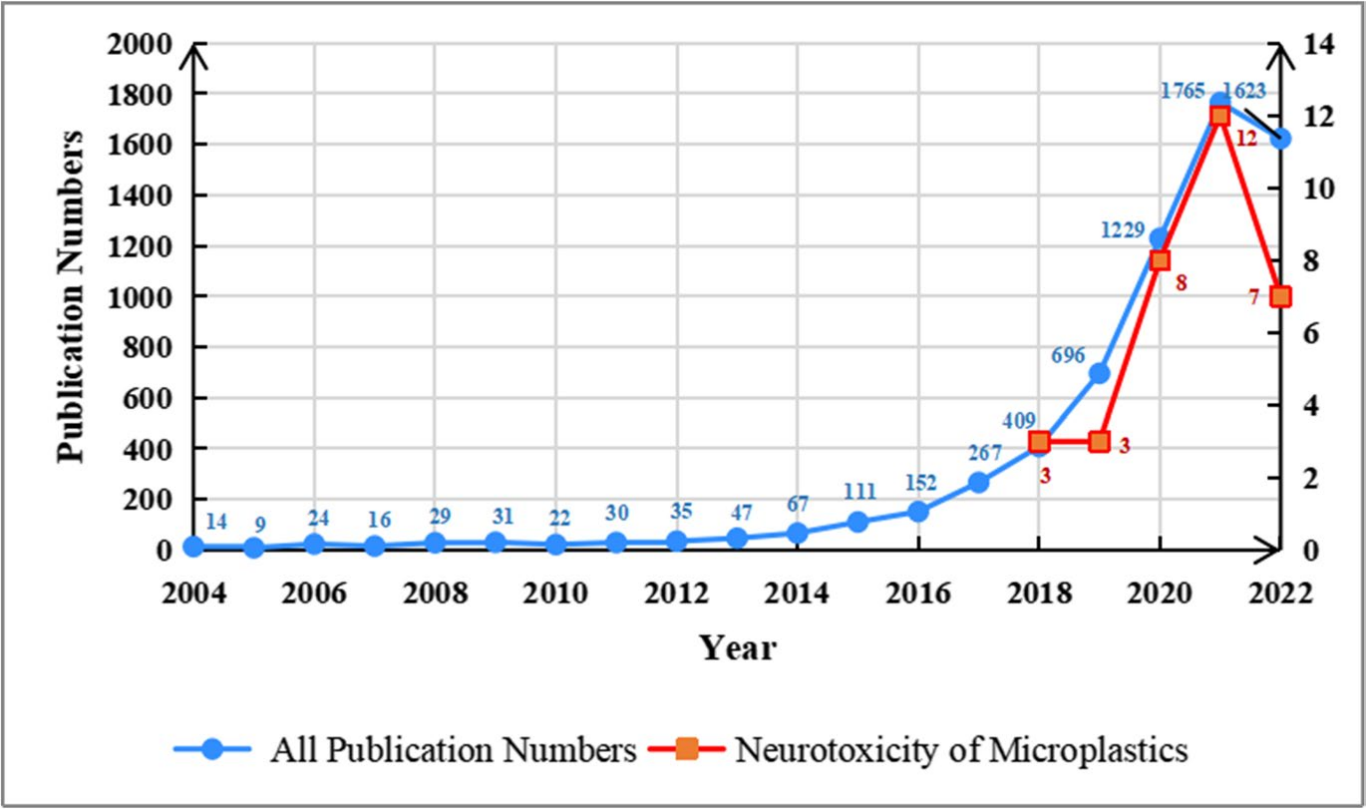
Distribution of articles published on microplastics and neurotoxicity studies over time, 2018–2022

Since 2004, the number of articles on microplastics has been on an upward trend and has seen a rapid rise in the last decade, with the fastest growth in articles occurring from 2018 to 2019 (growth rate: 76.58%). Neurotoxicity studies related to microplastics began in 2018, and the research trend follows the general trend of microplastics research, but the area represents only a very small portion of studies (the percentages of microplastic neurotoxicity studies of total microplastic studies from 2018 to 2022 are 0.73%, 0.43%, 0.65%, 0.68%, and 0.43%, respectively). See Fig. 1.

### Author collaboration and affiliation analysis

**Fig. 2:**
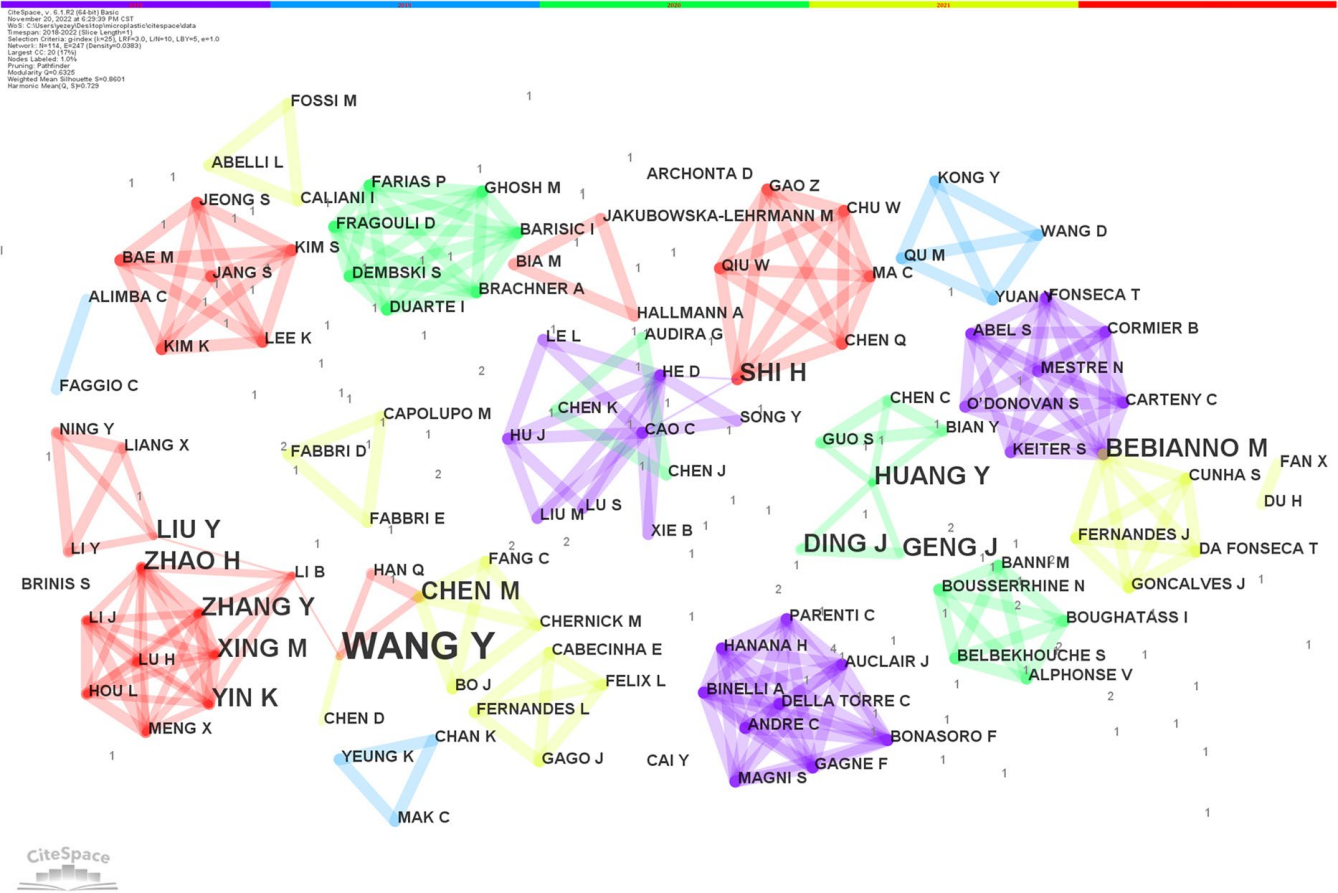
Author collaboration in microplastic neurotoxicity studies

As shown in Table 1, of the top 9 authors in the collaboration ranking (existence juxtaposition), 8 authors are from China and 1 is from Portugal. One of the more obvious collaborations occurred between Li, BY of Northeastern Forestry University in China and Bebianno, MJ of the University of the Algarve in Portugal. See Fig. 2.

**Table 1:** Microplastic neurotoxicity study author collaborations and their affiliations

Rank	Author	Centrality	Publications	Institution	Year^*^
1	Li B	0.04	1	Northeast Forestry Univ	2022
2	Wang Y^**^	0.03	4	Shanghai Ocean Univ etc.^***^	2021
3	Chen M	0.02	2	Cent China Normal Univ	2021
4	Liu Y	0.02	2	Northeast Forestry Univ	2022
5	Shi H	0.01	2	East China Normal Univ	2018
5^****^	Bebianno M	0.01	2	Univ Algarve	2018

^*^Time of the first appearance of cooperation

^**^CiteSpace merged authors, including Wang, YJ, Wang, Y, Wang, YH, and Wang, YY

^***^Including Shanghai Ocean University, Northeast Forestry University, Hohai University, and Huazhong Normal University

****Juxtaposition Sorting Fifth

**Fig. 3:**
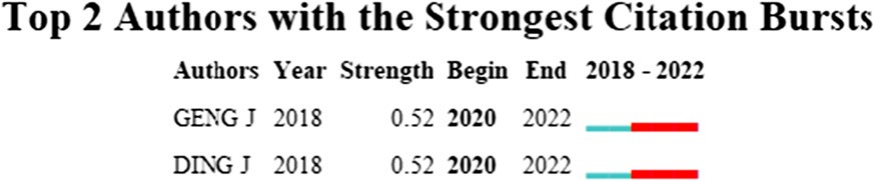
Collaborative bursting of microplastic neurotoxicity study authors. Parameter γ = 0.1, minimum duration of 2 years

The results of the burstness analysis showed that Geng, JJ at Nanjing University, China, and Ding, JN at Jiangnan University, China, were completed a prominent collaboration from 2020 to 2022. See Fig. 3.

### Cooperation and quantitative analysis of countries

**Fig. 4:**
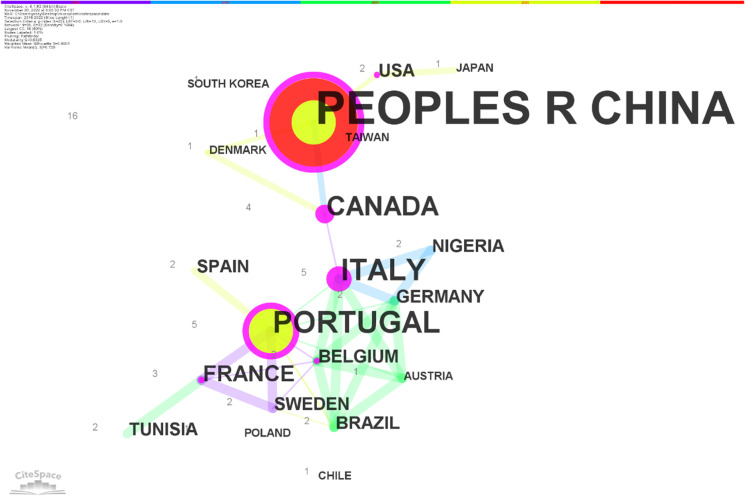
Countries and their collaborations in microplastic neurotoxicity studies

As shown in Table 2, microplastic neurotoxicity studies varied by country, with a total of 20 countries worldwide having published relevant studies. The analysis of country cooperation also reveals that the top three countries in terms of the number of publications in neurotoxicity studies of microplastics were China (16), Portugal (5), and Italy (5). The top three countries with the most cooperation with other countries is Italy (centrality: 0.63), Canada (centrality: 0.51), and Portugal (centrality: 0.31). During 2021, there was cooperation between China and the USA and Denmark; between Portugal and Spain and Brazil; between the USA and Japan; between Canada and Denmark; and between Brazil and Sweden. See Fig. 4.

**Table 2:** Top 5 countries in the world for microplastic neurotoxicity research

Ranking	Country	Frequent	Centrality	Year
1	People’s R China	16	0.30	2018
2	Portugal	5	0.31	2018
3	Italy	5	0.63	2018
4	Canada	4	0.51	2018
5	France	3	0.16	2018

### Keyword cooccurrence and clustering

**Fig. 5:**
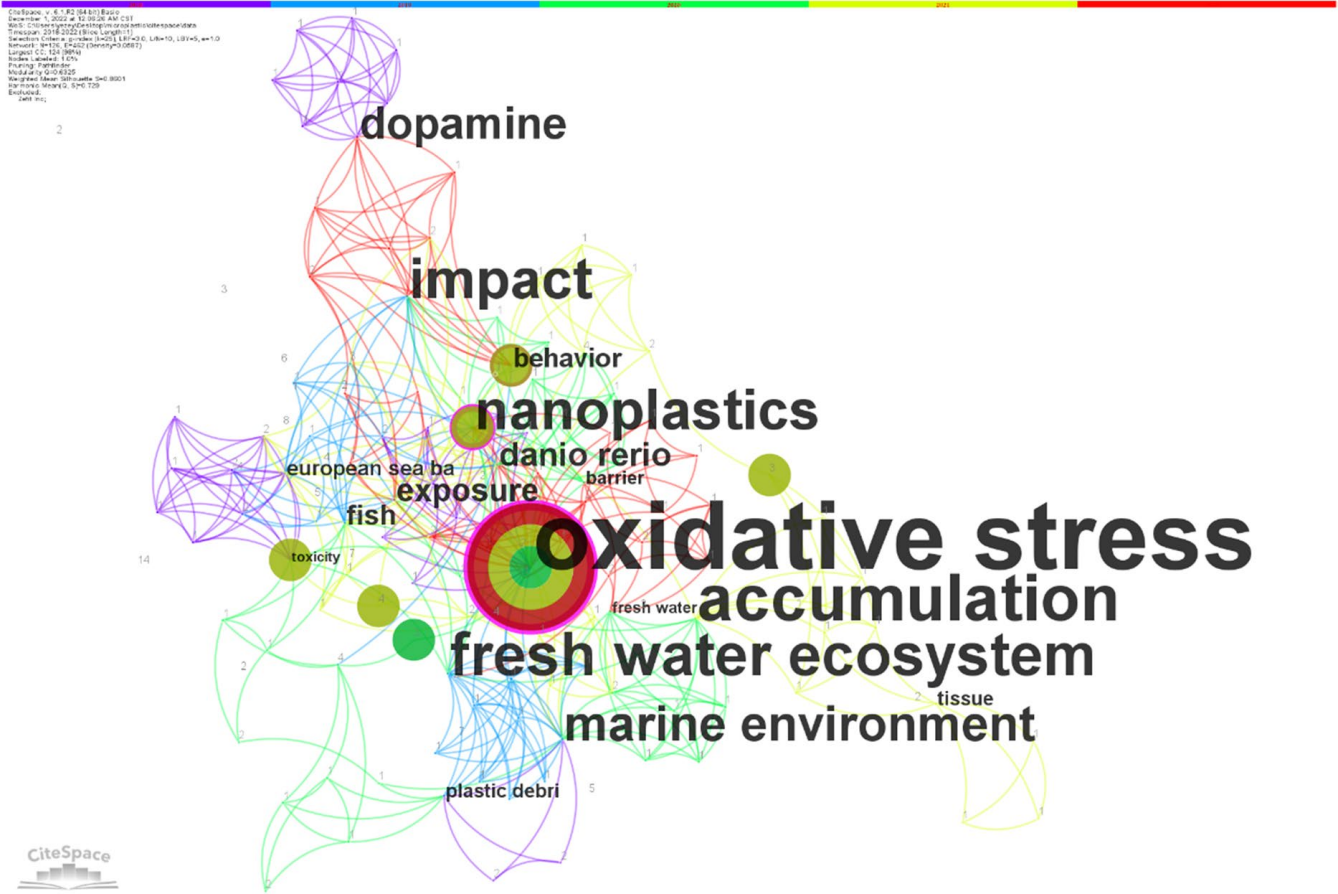
Cooccurrence of keyword associations in publications on microplastic neurotoxicity

As shown in Table 3, excluding the search keywords “microplastic” and “toxicity,” the top five keywords were “oxidative stress,” “nanoplastics,” “marine environment,” “behavior,” “fish,” “exposure,” and “plastic debris.” All of the above keywords had a strong centrality values of greater than 0.1 and these words were found to be central to the research. In addition to the above keywords, keywords with centrality values of greater than 0.1 include “accumulation,” “zebrafish,” “effects,” “dopamine,” and “fresh water ecosystem,” all of which play an important role in the study of the neurotoxicity of microplastics. See Fig. 5.

**Table 3:** Five most frequent keywords in microplastic neurotoxicity research articles

Rank	Keyword	Frequent	Centrality	Year
1	Oxidative stress	14	0.46	2018
2	Nanoplastics	8	0.27	2019
3	Marine environment	7	0.24	2018
4	Behavior	6	0.13	2019
5	Fish	5	0.14	2019
5	Exposure	5	0.15	2019
5	Plastic debris	5	0.1	2018

**Fig. 6:**
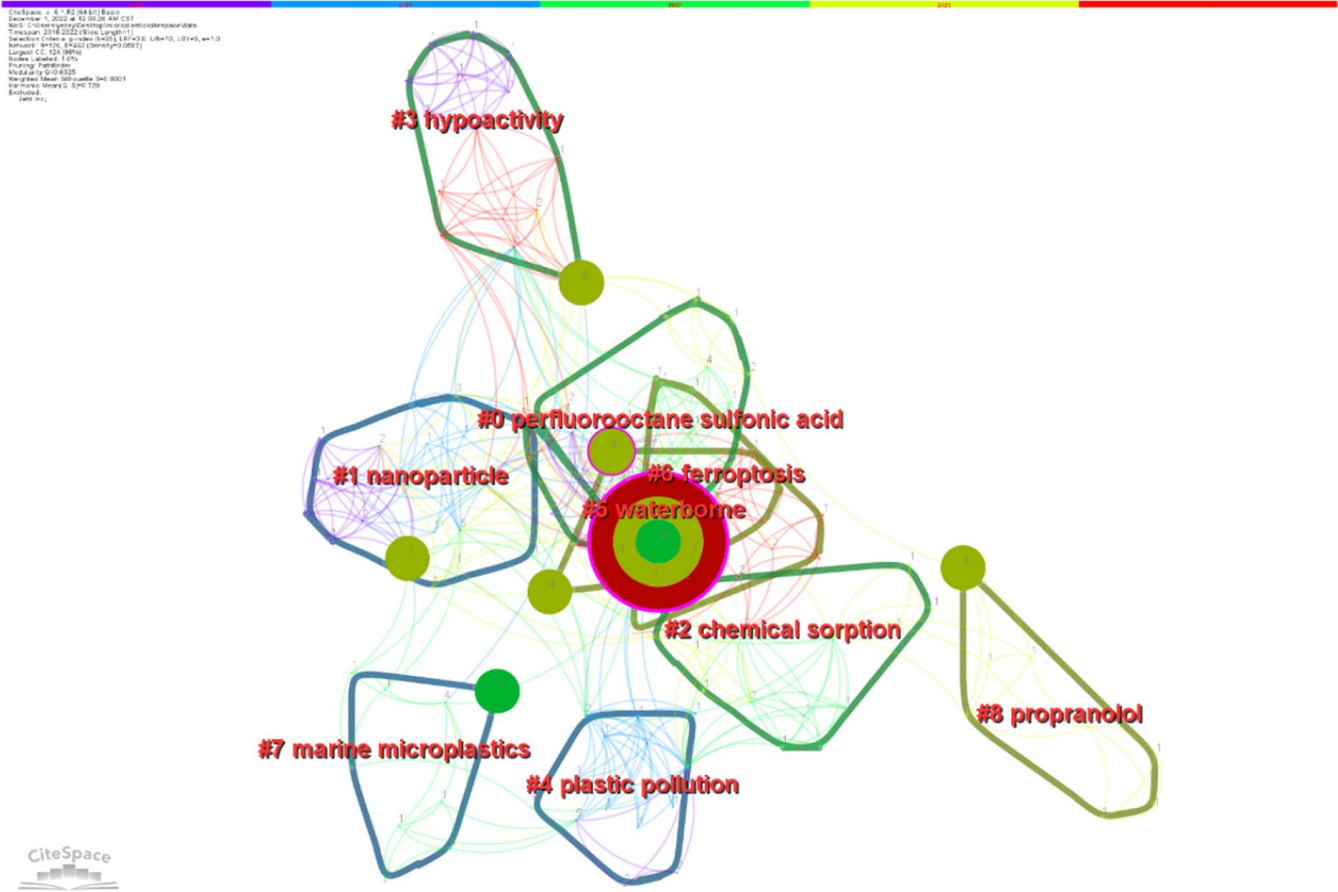
Clustering of keywords in publications on microplastic neurotoxicity

As shown in Table 4, a total of nine clusters were obtained, and the silhouette values were all greater than 0.7, with good clustering effects. The cluster with the most keywords is #0, which contains the main keywords “perfluorooctane sulfonic acid,” “polystyrene microplastic,” “*Eisenia fetida*,” “behavior test,” and “*Scrobicularia plana*.” During the 2022 time period, the keywords studied are mainly found in clusters 3, 5, and 6, which are represented by the keywords “hypoactivity,” “waterborne,” and “ferroptosis,” respectively. See Fig. 6.

**Table 4:** Summary of the eight largest clusters, showing size, silhouette, and representative terms to characterize each cluster

Cluster ID	Size	Silhouette	Mean (year)	Representative terms (LLR)	Keywords
#0	21	0.795	2019	Perfluorooctane sulfonic acid	Perfluorooctane sulfonic acid; polystyrene microplastic; *Eisenia fetida*; behavior test; *Scrobicularia plana*
#1	20	0.825	2018	Nanoparticle	Nanoparticle; microplastic pollution; oxidative stress; neurotoxicity; zebrafish *Danio rerio*
#2	15	0.818	2020	Chemical sorption	Chemical sorption; *Donax trunculus*; accumulation; embryotoxicity; bivalves
#3	15	0.931	2019	Hypoactivity	Hypoactivity; electroencephalogram; neurotransmitter; oxidative stress; microplastics
#4	14	0.929	2018	Plastic pollution	Plastic pollution; systemic toxicity; molecular mechanisms; marine environment; toxicological impacts
#5	12	0.874	2021	Waterborne	Waterborne; polyethylene microplastics; triphenyl phosphate; marine medaka (*Oryzias melastigma*); food chain
#6	10	0.849	2021	Ferroptosis	Ferroptosis; glutamine; cerebellum; liver-brain axis; microplastic
#7	9	0.843	2019	Marine microplastics	Marine microplastics; ecotoxicology; coastal pollution; Raman microspectroscopy; painted comber
#8	8	0.957	2021	Propranolol	Propranolol; sulfamethoxazole; integrated biomarker response; transcriptome; ultraviolet irradiation

### Time zone view and association of keywords

**Fig. 7:**
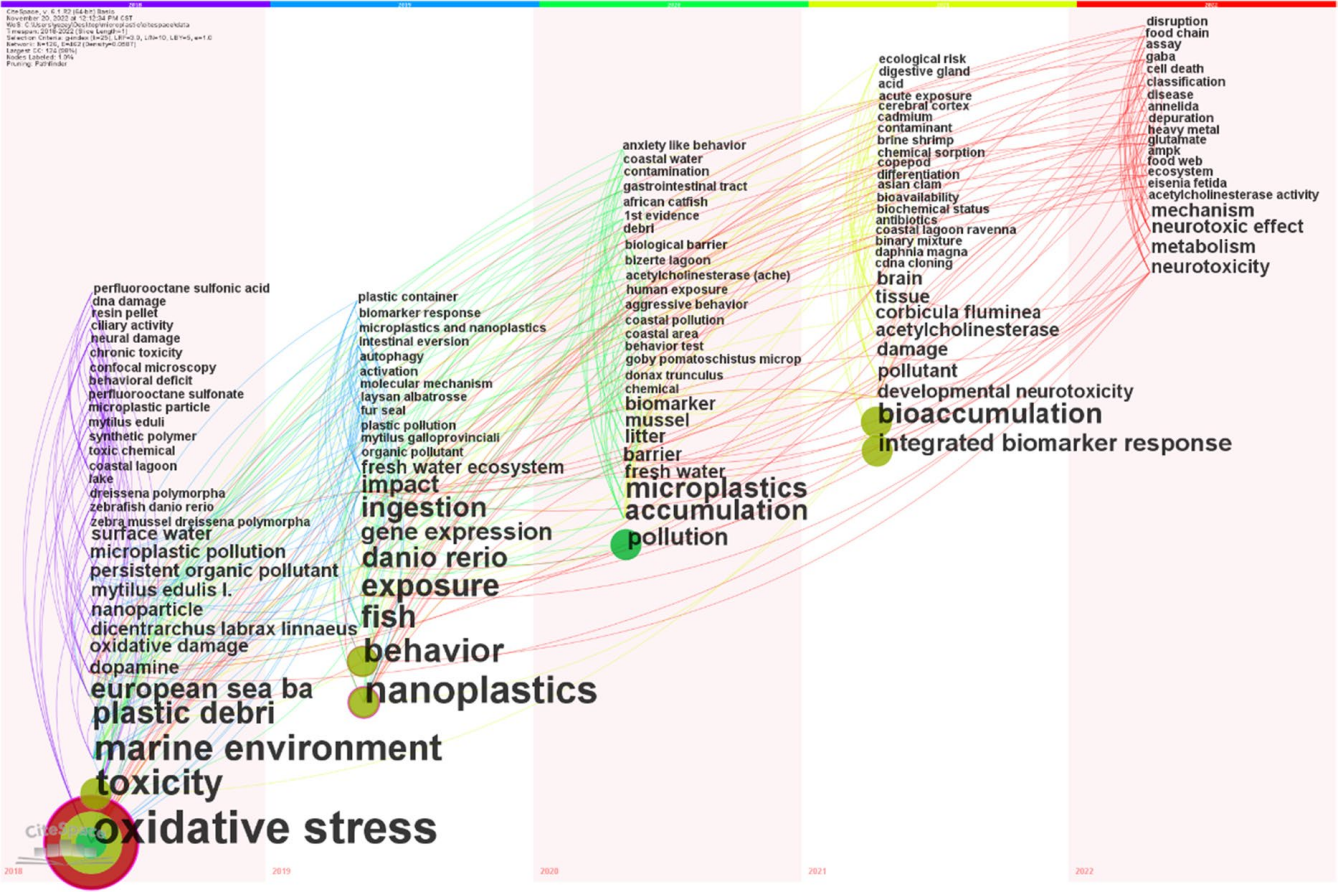
Time zone view and association of keywords for publications on microplastic neurotoxicity

Up to 31 keywords appear for the first time in microplastic neurotoxicity studies in 2018 and 20 appear in 2022. In 2022, “mechanism,” “neurotoxic effect,” “metabolism,” and “neurotoxicity” appeared more frequently than other keywords. Additionally the association between keywords appears not only within the same time zone, but also between keywords from different time zones. See Fig. 7.

### Cocited literature analysis

As shown in Table 5, the publication “Microplastics cause neurotoxicity, oxidative damage and energy-related changes and interact with the bioaccumulation of mercury in the European seabass” had a total of 11 counts among the top five cocitations in the field, while the rest had 8 counts. Three of the cocited literature burst analyses showed a burst trend for the 2020–2022 period.

**Table 5:** Top 5 cocited references and article sources for microplastic neurotoxicity research articles

Rank	Title	Frequency	Centrality	Year	Source journal
1	Microplastics cause neurotoxicity, oxidative damage and energy-related changes and interact with the bioaccumulation of mercury in the European seabass	11	0.3	2018	*Aquat Toxicology*
2	Quantitative investigation of the mechanisms of microplastics and nanoplastics toward zebrafish larvae locomotor activity	8	0.12	2017	*Science of the Total Environment*
3	Uptake and accumulation of polystyrene microplastics in zebrafish (*Danio rerio*) and toxic effects in liver	8	0.17	2016	*Environmental Science & Technology*
4	Accumulation, tissue distribution, and biochemical effects of polystyrene microplastics in the freshwater fish red tilapia (*Oreochromis niloticus*)	8	0.08	2018	*Environmental Pollution*
5	Microplastics effects in *Scrobicularia plana*	8	0.12	2017	*Marine Pollution Bulletin*

### Cocited literature burst analysis

**Fig. 8:**
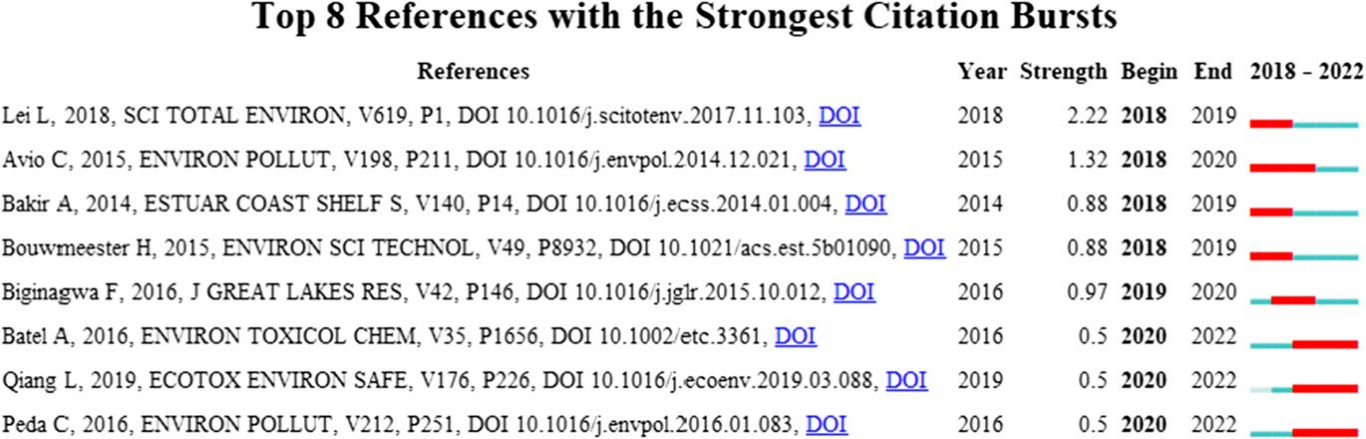
Cocited references on the neurotoxicity of microplastics from the literature

The study published by Batel A, Qiang L, and Peda C in 2020–2022 has strong cocited status. See Table 6 and Fig. 8.

**Table 6:** Bursting of cocited references in publications on the neurotoxicity of microplastics

No.	Title	Frequency	Centrality	Year	Source journal
1	Transfer of benzo[a]pyrene from microplastics to *Artemia* nauplii and further to zebrafish via a trophic food web experiment: CYP1A induction and visual tracking of persistent organic pollutants	4	0.18	2016	*Environmental Toxicology and Chemistry*
2	Exposure to microplastics decreases swimming competence in larval zebrafish (*Danio rerio*)	4	0.04	2019	*Ecotoxicology and Environmental Safety*
3	Intestinal alterations in European sea bass *Dicentrarchus labrax* (Linnaeus, 1758) exposed to microplastics: preliminary results	4	0.02	2016	*Environmental Pollution*

### Keyword burst analysis

**Fig. 9:**
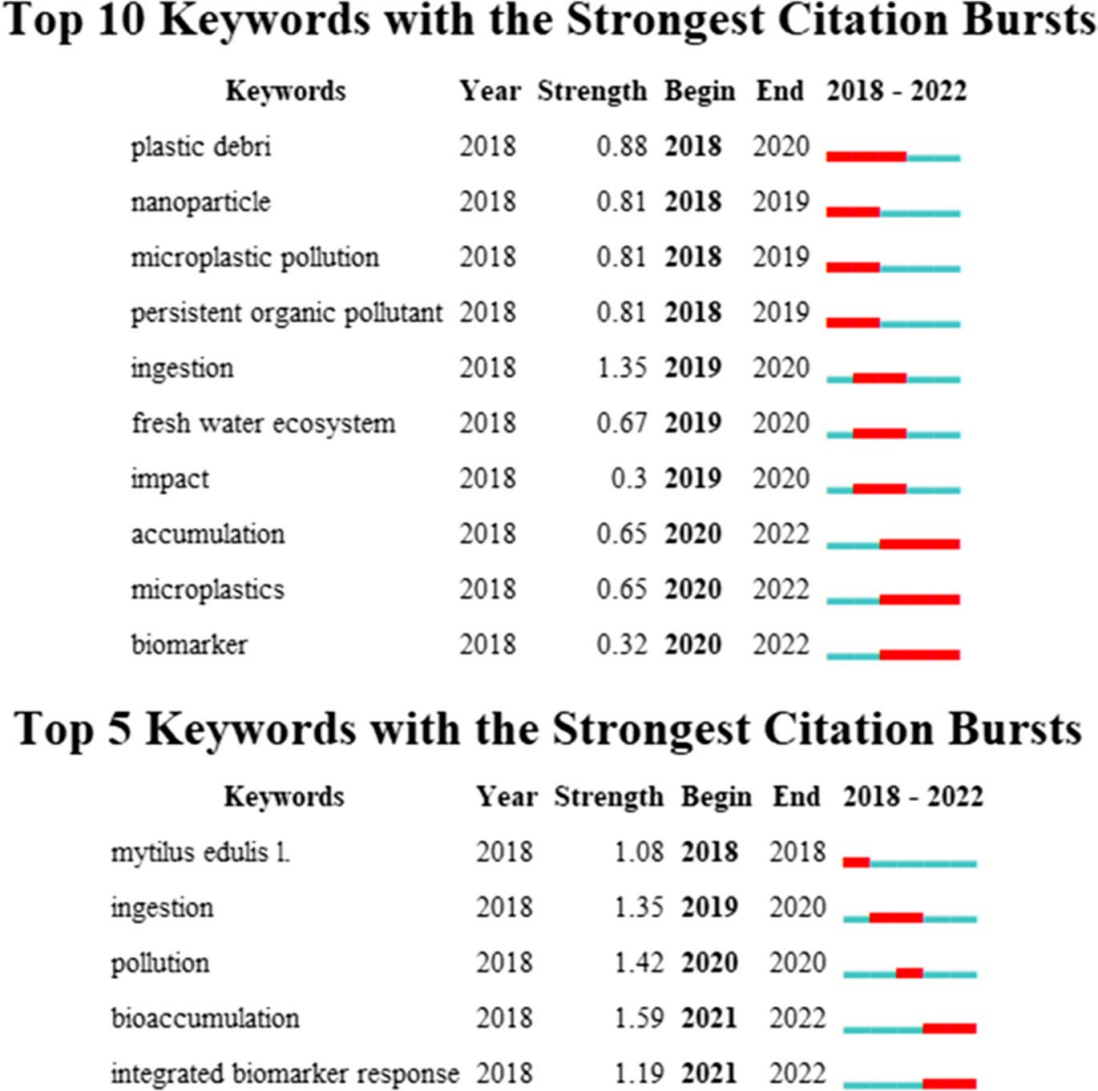
Bursting of keywords in publications on the neurotoxicity of microplastics. γ = 0.165, minimum durations of 2 years and 1 year

As shown in Fig. 9, after adjusting for the minimum duration, we find that when using 2 years as the minimum duration, in addition to the topic of “microplastics,” the research on “accumulation” and “biomarker” of the neurotoxicity of microplastics are in the trend of burst studies, from 2020 to 2022. The burst results were compared with a minimum duration of 1 year and identified “bioaccumulation” and “integrated biomarker response” as burst topics.

## Discussion

### Author collaboration analysis

Cooccurrence between authors represents a collaborative relationship between different authors, and author-to-author collaboration is a typical way of doing research today. Currently, with increasing specialization in the scientific research, an increasing number of researchers are choosing to collaborate and work together on collaborative publications to remove professional barriers (Cisneros et al., 2018). By counting the affiliations to which the authors belonged, it was found that most of the author collaborations occurred within the same institution or in different universities or institutes of related specialties in the same country. At the same time, because studies on the neurotoxicity of microplastics have been conducted only recently, there is relatively little collaboration among authors (centrality < 0.1). The rest of the authors were relatively independent of each other, and future studies could strengthen collaboration between authors and break down the knowledge barriers.

### Author collaboration burst analysis

Geng, JJ from Nanjing University, China, and Ding, JN from Jiangnan University, China, were found to be involved in cooperative bursts from 2020 to 2022 from the authors’ burst analysis. Together, the authors explored the harmful effects of microplastics of different sizes on red tilapia and found that the toxicity of microplastics may not be simply monotonically negatively correlated with their size. Furthermore, in a follow-up study comparing the interaction of aging microplastics with the antibiotic sulfamethoxazole (SMX) and β-blocker propranolol (PRP), using red tilapia as a model, the authors concluded that aging of microplastics may lead to more complex changes in aquatic organisms due to their interaction with drugs (Ding et al., 2020; Huang et al., 2021). Because of their collaborative burst, there is reason to believe that the two scholars will continue to collaborate and contribute to the development of research in the field for some time to come.

### Keyword cooccurrence analysis

Keywords are usually considered to reflect the research direction and theme of an article and are a condensation and distillation of the core content of an article. It is also possible to identify emerging trends in the research field based on the analysis of certain features of the published literature (e.g., keywords) (Li et al., 2022). The zebrafish has been an important animal model in the field of neuroscience since the 1970s, and its many attributes, such as its high vitality and seasonal reproduction, have made it one of the most important organisms in toxicology, developmental, and behavioral research (Spence et al., 2008; Vascotto et al., 1997). The marine environment is an important environment for human survival. Microplastics and plastic debris are continuously discharged into the ocean, and plastics account for 60–80% of the land-based waste entering the marine environment (Derraik, 2002), and pass through the marine ecosystem into invertebrates and other organisms, causing various toxicological effects (Alimba & Faggio, 2019; Gall & Thompson, 2015). Polystyrene induces pathways of oxidative stress (Lei et al., 2018; Lu et al., 2016). The mechanism involved is the formation of reactive oxygen species (ROS) through the formation of excess O/N molecules, which, when produced in excess, inhibit the antioxidant system and cause damage to lipids, proteins, DNA, and other structures by altering the physiological homeostasis of cells (Alimba & Faggio, 2019). Nanoplastics (NPs) are defined as synthetic polymer particles with particle sizes in the range of 1–1000 nm. However, Ding et al. concluded that the toxicity of N/MPs to red tilapia could not be monotonically concluded simply by size from different sizes of microplastics in their experiments. The toxic effects of different sizes of microplastics still need further study (Ding et al., 2020). Adult zebrafish are often used for behavioral testing because their physiology is similar to that of humans. Mak et al. summarized the characteristics of behavioral changes in zebrafish after microplastic contamination in previous studies, including decreased physiological functions in terms of drowsiness, anxiety, physical-motor activity, food intake, reproductive performance, exploratory activities, and social interactions (Mak et al., 2019).

### Temporal distribution and evolution of keywords

A time zone distribution map of keywords is used to track the focus of research within a time range by dividing it into different time intervals (Wu et al., 2021). In this way, we can observe changes in the focus of research on the neurotoxicity of microplastics over time. While the concept of microplastics was introduced in 2004, a literature search of relevant topics showed that targeted studies on the neurotoxicity of microplastics started in 2018. Therefore, the time zone diagram shows that in 2018, the effects of microplastics of different sizes and forms, such as “neural damage,” “toxicity” and “nanoparticle,” “plastic debris,” and “synthetic polymer,” were proposed as effects of “persistent organic pollutant” to study the effects of microplastics on organisms. The environments studied mainly include “marine environment,” “coastal lagoon,” “lake,” and “surface waters.” The main objects of the study include “European sea bass,” “*Mytilus*,” and “zebrafish.” Effect indicators of interest include “oxidative stress,” “dopamine,” “DNA damage,” and “ciliary activity,” and whether the adsorption of “perfluorooctane sulfonic acid” and microplastics has a “chronic toxicity” effect and “behavioral deficit” effect on nerves.

In 2019, microplastic research focused more on “ingestion,” “behavior,” “exposure,” “gene expression,” and the “freshwater ecosystem” than in the previous year. “*Danio rerio*” also appears more frequently as a keyword in research. “Biomarker response,” “activation,” “autophagy,” and “molecular mechanism” have also come to the forefront of scholars’ attention. At the same time, “intestinal eversion” and other gastrointestinal and intestinal effects and “plastic container” were first reported in relation to the neurotoxic effects of microplastics.

In 2020, the study microplastic “pollution,” “accumulation,” and “biomarker” was given more attention, and the number of marine organisms studied was expanded. In response to behavioral manifestations, “aggressive behavior” and “anxiety-like behavior” appear. Sarasamma et al. demonstrated that high concentrations of microplastics or nanoplastics can alter the temperament of adult zebrafish to make them aggressive, among other behaviors (Sarasamma et al., 2020). At the same time, for the first time, “biological barrier” and “acetylcholinesterase (Ache)” were studied to extend mechanisms of the neurotoxicity of microplastics. Acetylcholinesterase activity as a common indicator of neurotoxicity. Urban-Malinga et al. found a 60% reduction in acetylcholinesterase activity in polychaetes after exposure to environmentally relevant microplastic concentrations compared to controls (Urban-Malinga et al., 2022).

In 2021, “bioaccumulation” was added as a keyword in research, and “acute exposure” appeared for the first time in the study of the neurotoxicity of microplastics. In addition to the previous year’s biomarker study, the first study on “integrated biomarker response” and “biochemical status” and “differentiation” was conducted. At the same time, the emergence of “binary mixture” and “cadmium” indicates that the toxic effects of a single microplastic were explored, as well as the interaction and joint effects of multiple substances. The results of Miranda et al. found that microplastics modulate the sublethal toxic effects of Cd on wild microsomal larvae, especially the neurotoxicity. In addition, the living environment of juvenile fish before development affects the sublethality of Cd, microplastics, and their mixtures to the organisms and suggests that the effects on humans need further study (Miranda et al., 2019). To clarify neurotoxicity studies, “developmental neurotoxicity” and “cerebral cortex” emerged in 2021. For the first time, we were able to study the effects of “bioavailability” and “antibiotics,” and to use the “cDNA cloning” method in this field.

In 2022, more studies were conducted on “neurotoxicity,” “neurotoxic effect,” “mechanism,” and “metabolism.” Acetylcholinesterase has appeared for three consecutive years, and the object of study shifted from aquatic organisms to “Annelida” and “*Eisenia fetida*.” At the same time, keywords “food chain,” “food web,” “ecosystem,” “depuration,” and “assay” were used for the first time in the study of neurotoxicity, which is in line with the larger study of microplastics. The emergence of “ampk,” “glutamate,” and “gaba” indicates that research on the mechanisms of the neurotoxicity of microplastics will continue to be strengthened. At the same time, “cell death” and “disruption” were introduced for the first time, and the research on “heavy metal” was continued from the previous stage. Heavy metals were widely used as important catalysts in the plastic manufacturing industry, and they can be found in the living environment such as soil together with microplastics and stored for a long time (Carbery et al., 2018). At the same time, microplastics, due to their special surface structure and functional groups, can adsorb harmful substances in the surrounding environment and form complex pollutants that can cause damage to human health through the food chain and food web (Liang et al., 2022). Therefore, it is necessary to strengthen the research on the interaction between heavy metals and microplastics in living environment and their toxicity in the future.

### Cocited reference burst analysis

An analysis of three papers included in a cocited burst for 2020–2022 revealed Batel et al.’s study establishing a food chain of haloarchaea sessile larvae and zebrafish to analyze the transfer of microplastics and related persistent organic pollutants between different trophic levels, creating a model of microplastic aggregation in food webs ending with a vertebrate model (Batel et al., 2016). Qiang et al. investigated the effects of microplastics on zebrafish embryos and larvae and found that exposure to microplastics resulted in an upregulated expression of genes related to inflammation and oxidative stress and significantly reduced swimming ability in zebrafish larvae (Qiang & Cheng, 2019). For the first time, Pedà et al. investigated the intestinal response of European seabass after long-term ingestion of microplastics and found that microplastics had the greatest effect on pathological changes in the distal intestine. Furthermore, exposure and damage show a clear time-response relationship and emphasize the impact of microplastic pollution on marine trophic networks (Pedà et al., 2016).

### Keyword burst analysis and future research directions

A keyword burst analysis can identify keywords that do not reach the research threshold today but have research prospects for future development, and thus grasp the development of the theme of a research field (Chen et al., 2012). Based on the results obtained by adjusting the minimum duration, it is reasonable to assume that the focus of current and future neurotoxicity studies centers on accumulation in organisms leading to neurotoxic effects and a combined response to biomarkers. In a discussion of the future of microplastics research, Da Silva Brito et al. suggested that enhanced longitudinal monitoring of microplastics and the development of chronic modeling studies are needed to help clarify the organismal damage caused by long-term exposure and accumulation of microplastics (Da Silva Brito et al., 2022). Meanwhile, the accumulation effect is reflected not only in the accumulation of microplastics in the body, but also in the accumulation of drugs in the body. Huang et al. investigated the interaction between sulfamethoxazole and propranolol in aged and unprocessed microplastics and found that aging of microplastics increased propranolol accumulation in the brain by 82.3%. It was also demonstrated by combining the responses of various biomarkers under various exposure conditions that the combined biological benefits caused by the interaction of microplastics with drugs change with the aging of microplastics (Huang et al., 2021). This provides new ideas and directions for future research.

## Limitations

Although the WoSCC database is widely recognized, there are some literatures in other data platforms remains. The reason for this is that some databases (e.g., PubMed) are limited in the use of CiteSpace software, which can be addressed in the future when the relevant analysis software is updated.

## Conclusion

Microplastic neurotoxicity studies are currently on the rise. Results show that the highest number of publications are from China, collaboration is most common between Italy and other countries, and less collaboration occurs between authors. The focus of future research on the neurotoxicity of microplastics may revolve around “accumulation” and “integrated biomarker response.”

## Data Availability

The original data used in this study are all publicly available data from the Web of Science Core Collection.

## Declarations

All authors have read, understood, and have complied as applicable with the statement on “Ethical responsibilities of Authors” as found in the Instructions for Authors and are aware that with minor exceptions, no changes can be made to authorship once the paper is submitted.
